# Early life stress-induced alterations in rat brain structures measured with high resolution MRI

**DOI:** 10.1371/journal.pone.0185061

**Published:** 2017-09-25

**Authors:** R. Angela Sarabdjitsingh, Manila Loi, Marian Joëls, Rick M. Dijkhuizen, Annette van der Toorn

**Affiliations:** 1 Department Translational Neuroscience, Brain Center Rudolf Magnus, University Medical Center Utrecht, Utrecht University, Utrecht, The Netherlands; 2 University of Groningen, University Medical Center Groningen, Groningen, The Netherlands; 3 Biomedical MR Imaging and Spectroscopy Group, Center for Images Sciences, University Medical Center Utrecht, Utrecht University, Utrecht, The Netherlands; Klinikum der Johann Wolfgang Goethe-Universitat Frankfurt, GERMANY

## Abstract

Adverse experiences early in life impair cognitive function both in rodents and humans. In humans this increases the vulnerability to develop mental illnesses while in the rodent brain early life stress (ELS) abnormalities are associated with changes in synaptic plasticity, excitability and microstructure. Detailed information on the effects of ELS on rodent brain structural integrity at large and connectivity within the brain is currently lacking; this information is highly relevant for understanding the mechanism by which early life stress predisposes to mental illnesses.

Here, we exposed rats to 24 hours of maternal deprivation (MD) at postnatal day 3, a paradigm known to increase corticosterone levels and thereby activate glucocorticoid receptors in the brain. Using structural magnetic resonance imaging we examined: i) volumetric changes and white/grey matter properties of the whole cerebrum and of specific brain areas; and ii) whether potential alterations could be normalized by blocking glucocorticoid receptors with mifepristone during the critical developmental window of early adolescence, i.e. between postnatal days 26 and 28.

The results show that MD caused a volumetric reduction of the prefrontal cortex, particularly the ventromedial part, and the orbitofrontal cortex. Within the whole cerebrum, white (relative to grey) matter volume was decreased and region-specifically in prefrontal cortex and dorsomedial striatum following MD. A trend was found for the hippocampus. Grey matter fractions were not affected. Treatment with mifepristone did not normalize these changes.

This study indicates that early life stress in rodents has long lasting consequences for the volume and structural integrity of the brain. However, changes were relatively modest and–unlike behavior- not mitigated by blockade of glucocorticoid receptors during a critical developmental period.

## Introduction

Early life stress (ELS) refers to a condition of prolonged stress exposure (single or multiple stressors) during the perinatal period that exceeds the child’s coping resource [1]. It is well established that adverse experiences early in life have an enduring impact on the neuroendocrine system, and are associated with impaired memory and cognitive function, both in humans and rodents [2–4]. In humans, these alterations may increase the risk to develop psychopathology later in life [4–6]. Stress-related psychopathology is characterized by anomalies in the structural integrity of the brain [7,8]. These may have started already early in life, as a consequence of adversity. In agreement, structures of key areas implicated in the stress response as well as in the processing of higher cognitive function, e.g. the prefrontal cortex (PFC) [9] and hippocampus [10], are typically compromised by childhood adverse experiences. In rodents as well as humans, structural changes comprise diminished hippocampal volume [11,12], reduced dendritic length and branching of neurons within hippocampal regions [13,14], smaller prefrontal cortex volume [15,16], and anomalies in dendritic morphology of striatal subregions as well striatal volumetric reduction [17,18]. Moreover, adolescents who as a child experienced severe institutional deprivation showed greater amygdala volume [19]. In line with this, results from human neuroimaging studies reported an association between early life stress and alteration in the white and grey matter volume [20,21], also in areas involved in stress regulation [22].

The driving force behind the structural anomalies after early life stress may be the functionality of the hypothalamus-pituitary-adrenal (HPA) axis. This axis is known to be disrupted by adverse early life experiences [23]. The HPA axis is the regulatory system for stress responsivity and a deregulation, resulting in increased corticosterone levels, may alter the structural and functional plasticity of the developing brain [4,24]. Based on this notion, we hypothesized that blocking the effects of circulating corticosterone acting through glucocorticoid receptors (GRs) may attenuate the early life stress-related brain structure anomalies.

To test this idea, male rats were exposed to 24 hours of maternal deprivation (MD) at postnatal day (PND) 3. This represents a severe model of maternal neglect that (at PND 3) induces a substantial release of corticosterone [25]. In rodents, brain areas involved in higher cognitive function (e.g. prefrontal cortex and hippocampus) mainly develop postnatally; therefore they may be particularly vulnerable to the effects of early life stress. Our first aim was to examine if MD causes long lasting alterations in the normal brain development, using high resolution structural magnetic resonance imaging (MRI). Secondly, we assessed whether a transient blockade of GRs (at PND 26–28) can normalize the expected brain structural abnormalities. The choice of intervening during early adolescence is based on the high sensitivity of the brain and the HPA axis during this critical time window [26]. Moreover, brief administration of a glucocorticoid receptor antagonist during adolescence was recently found to ameliorate the behavioral deficits associated with early life stress [27].

## Materials and methods

### Experimental animals

Prior to the start of the study all animal procedures were approved by the local animal ethics committee at Utrecht University, the Netherlands. All efforts were made to minimize suffering. Adult male and female Wistar rats were purchased from Charles River (Sulzfeld, Germany) and habituated in random pairs to the animal facilities for two weeks. For breeding, male rats were put together with female rats in a 1:2 ratio for a period of 10 days. Females were housed in pairs after mating until the last week of pregnancy when they were housed individually. Every morning before 9 am cages were checked for births; upon birth that particular day was noted as postnatal day 0 (PND 0). Dams with litters were left undisturbed until PND 3 when culling took place. Litters contained on average 9 ± 1 pups, whenever possible evenly distributed over males and females. After culling, litters were randomly assigned to either the maternal deprivation (MD) condition or the control (no maternal deprivation; noMD) group. In the MD group, the mother was placed in a separate cage; the pups went back in the home cage and were placed on a heating pad (32°C) in a different room than where the mother was housed. MD litters were kept in this room for 24 h and then placed back with the dam, as described elsewhere [28]. All animals survived the MD procedure. NoMD litters were left entirely undisturbed.

On PND 21 the litters were weaned and housed in same-sex groups of either two or three. Only males were used for this experiment. The MD and noMD rats were randomly assigned to either mifepristone (MIF; 5mg /100g bodyweight) or vehicle (VEH) administration through oral gavage, twice daily (early morning and late afternoon) on PND 26, 27 and 28. Each rat received the mifepristone powder (Sigma-Aldrich Chemie B.V., Zwijndrecht, the Netherlands) dissolved in 15μL 99% ethanol mixed with 1mL coffee cream (Campina, Woerden, the Netherlands) or vehicle by an experimenter that was blind to the treatment group. To minimize litter effects, offspring from each MD and noMD litter were randomly divided between MIF and VEH groups. The cages with the experimental animals were randomly placed in the room and kept under standard housing conditions (dark/light phase 12:12, lights on at 8 a.m., humidity 55±15%, temperature 20–22°C), received food and water *ad libitum* and were weekly handled for 1 minute.

### Tissue preparation procedure

In order to obtain high signal-to-noise ratio and spatial resolution for the structural brain images [29], we conducted post-mortem MRI, for which three months’ old rats (n = 10 per group) were anaesthetized with pentobarbital sodium salt (0.5 ml i.p.) and transcardially perfused with saline (0.9% NaCl) followed by 4% paraformaldehyde (PFA) in phosphate buffer (PB; 0.1 M; pH 7.4). All extracranial tissue was removed and the brains were left in the skulls to minimize the potential risk of deformation. After overnight post-fixation at 4°C, the skulls containing the brains were stored in the refrigerator (4°C) in PB with 0.01% sodium azide until use for high-resolution post-mortem MRI.

### MRI acquisition

Post-mortem high-resolution structural MRI was performed on a 9.4 T horizontal bore MR system (Varian, Palo Alto, CA, USA) equipped with a 6 cm internal diameter (ID) gradient insert with gradients up to 1 T/m. A custom made solenoid coil with an internal diameter of 2.6 cm was used for excitation and reception of the MR signal. The perfusion-fixed brains were inserted with the skulls intact in a custom-made holder and immersed in a proton-free perfluoropolyether solution (Fomblin, Solvay Solexis). Diffusion tensor imaging (DTI) was performed using a 3D diffusion-weighted spin-echo sequence with an isotropic spatial resolution of 150 μm, where the read- and phase- encode directions were acquired using 8-shot Echo Planar Imaging (EPI) encoding and the second phase direction was linearly phase-encoded (repetition time (TR)/echo time (TE) 500/32.4 ms, 220*128*108 matrix, field-of-view (FOV) 33*19.2*16 mm^3^,Δ/δ 15/4 ms, b 3842 s/mm^2^, 60 diffusion-weighted images in non-collinear directions and 5 images without diffusion weighting (b = 0), number of averages 1, total number of images 65). Subsequently, four 3D Balanced Steady-State Free Precession (BSSFP) images were acquired with an isotropic spatial resolution of 100 μm (TR/TE 15.4/7.7 ms, flip angle 40°, 320*160*190 matrix, FOV 32*16*19 mm^3^, 6 averages, pulse angle shift 0°, 90°, 180° and 270°). Total acquisition time was 3 hours and 7 minutes.

### Morphometric analysis

**Fig 1:**
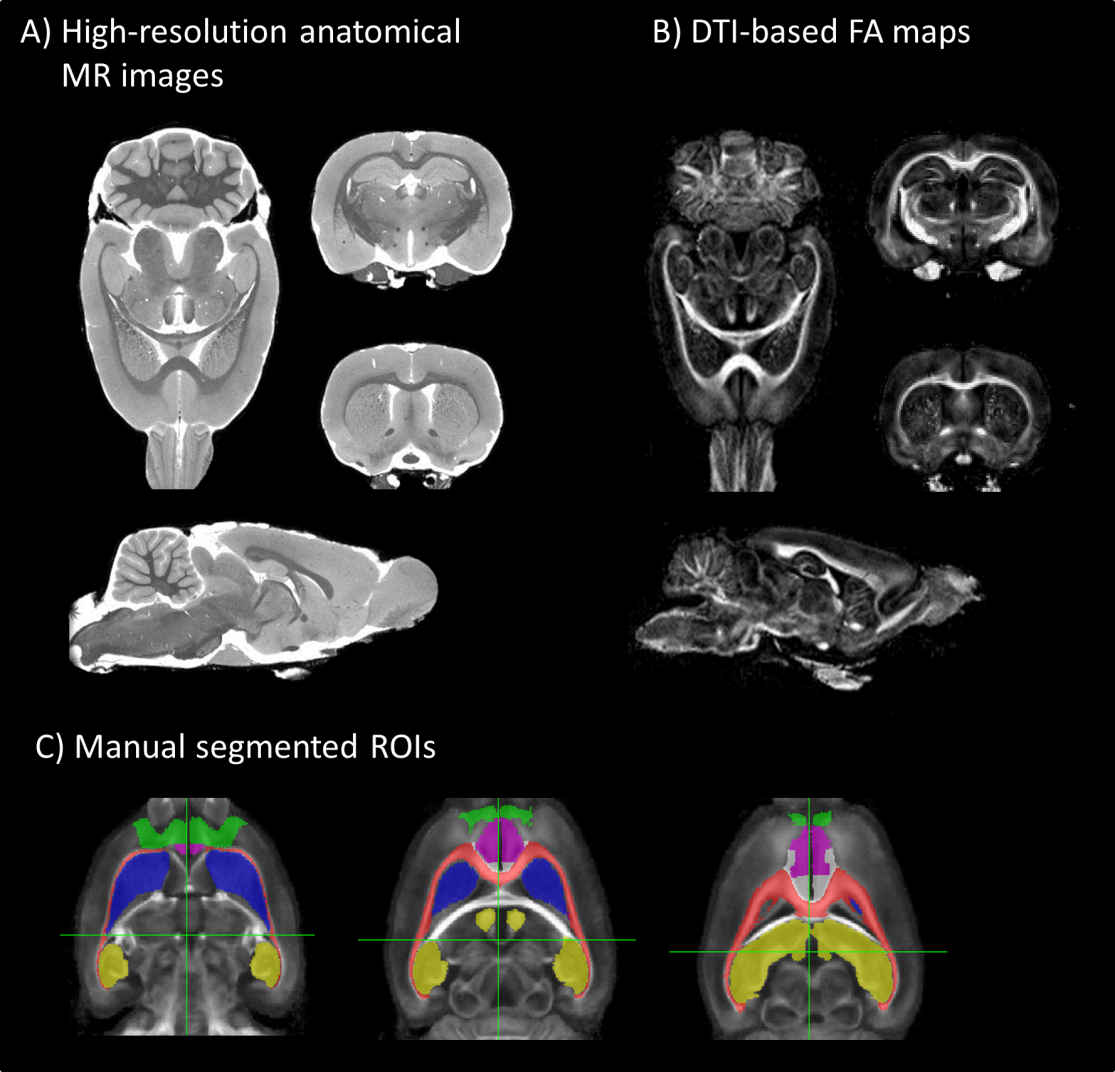
Representative MR images of the rat brain. A) High-resolution post-mortem anatomical images (100 x 100 x 100 μm^3^) of the rat brain template from all experimental animals (n = 40). Individual scans were registered to this template to perform voxel-wise comparisons on brain volume. B) Diffusion tensor imaging-based FA maps (150 x 150 x 150 μm^3^) of the rat brain template. The FA maps were used to analyze structural integrity properties of the white matter. C) ROIs, for example OFC (green), mPFC (purple), corpus callosum (red), dorsomedial striatum (blue) and hippocampus (yellow), depicted on the average rat brain template.

The four BSSFP images were added as complex images to obtain a single BSSFP image with reduced banding artifacts in the brain. To test whether maternal deprivation affected regional brain tissue volumes, the BSSFP images were used to obtain brain masks using Brain Extraction Tool 2 (BET2) as implemented in the FMRIB (Oxford Center for Functional MRI of the Brain) Software Library (FSL) [30]. Subsequently, a template image for morphometry analysis was iteratively computed by applying non-rigid registration of subject images to the current template using FMRIB’s Non-linear Image Registration Tool (FNIRT) after linear registration using FMRIB’s Linear Image Registration Tool (FLIRT) [31,32], both implemented in FSL. The template was generated by (1) non-rigid registration of the images of the noMD/vehicle group to the current template (starting with one image of this group), (2) averaging all the transformed images to generate a new template, (3) repeating the registration procedure for the new template. Representative images are shown in Fig 1A and 1B.

We used a region of interest (ROI)-based approach to assess volumetric and white/grey matter properties, as previously described [29]. The following ROIs were chosen based on their prominent role in the stress circuitry and sensitivity to ELS [9,10]: Amygdala, Hippocampus, dorsomedial Striatum (dmStriatum), Prefrontal Cortex (PFC) and its subregions medial PFC (mPFC), Orbitofrontal Cortex (OFC) and Insular Cortex. We also included the whole cerebrum, lateral ventricles and corpus callosum as ROI. The ROIs were drawn on the template image using an in-house developed rat brain atlas based on the stereotaxic atlas from Paxinos and Watson [33] and registered to the individual rat brain images; from this, the size of the ROI in the individual images was obtained (Fig 1C).

White and grey matter volumes in regions of interest were determined in the the FA and mean diffusivity maps. Pixels were assigned to white matter if their FA was higher than 0.25 and the mean diffusivity lower than 0.34*10–3 mm2/s, to grey matter if the FA was lower than 0.25 and the mean diffusivity lower than 0.34*10^−3^ mm2/s, and to CSF if the FA was lower than 0.25 and the mean diffusivity higher than 0.34*10^−3^ mm2/s.

### Statistical analysis

Data were analyzed using a two-way independent ANOVA. The rearing condition (MD or noMD) and intervention (VEH or MIF) were used as independent variables with p < .05 considered to be significant. Tukey’s posthoc test was used where applicable. Data is expressed as mean ± SEM. All statistical analyses were conducted using SPSS version 20.0 (IBM, United States).

## Results

### Volumetric analysis after early life stress

To assess whether MD and/or MIF treatment affected brain structure, we measured total volume of the cerebrum and individual ROIs, based on the high-resolution structural images (Fig 1A and 1C). The data are presented in Table 1. No significant volumetric changes of the whole cerebrum were found due to MD or MIF treatment. In most of the ROIs, analysis of total volume also did not reveal any changes. In the PFC however, MD significantly reduced volume (main MD effect F(1, 34) = 7.55, p < 0.01), which was not affected by MIF treatment. Subregion analysis of the PFC showed that this effect was mainly present at the level of the OFC and mPFC but not the insular cortex (Table 1). These data indicate that MD specifically reduces the volume of the PFC without affecting the total cerebral volume but that this reduction was not reversed by MIF treatment.

**Table 1:** Volumes of the different ROIs based on high-resolution anatomical MRI. N = 10 rats per group. Data represents mean ± SEM in μl and was analyzed using 2-way ANOVA with MD and mifepristone treatment as main factors. Statistical significance based on p-values for the main effect is presented in the last three columns: * p < 0.05, ** p < 0.01, ns = not significant.

	Vehicle		Mifepristone		MD	Treatment	Interaction
**Volume (μl)**	**No MD**	**MD**	**No MD**	**MD**			
**Cerebrum**	1550.5 ± 10.0	1546.6 ± 11.9	1573.8 ± 16.1	1548.9 ± 15.7	ns	ns	ns
**Corpus Callosum**	30.4 ± 2.2	31.3 ± 2.4	31.0 ± 2.2	30.5 ± 3.0	ns	ns	ns
**Lateral Ventricles**	40.1 ± 1.8	38.6 ± 0.7	39.6 ± 1.1	38.9 ± 1.3	ns	ns	ns
**Hippocampus**	91.0 ± 1.1	91.4 ± 0.7	91.3 ± 1.2	90.6 ± 1.4	ns	ns	ns
**Amygdala**	29.2 ± 0.3	29.1 ± 0.3	29.4 ± 0.3	29.6 ± 0.5	ns	ns	ns
**dmStriatum**	16.8 ± 0.2	17.0 ± 0.3	17.6 ± 0.5	16.6 ± 0.3	ns	ns	ns
**PFC**	101.7 ± 0.4	99.6 ± 0.3	101.4 ± 1.0	98.9 ± 1.1	**	ns	ns
*OFC*	22.9 ± 0.2	22.1 ± 0.1	22.4 ± 0.3	22.2 ± 0.2	*	ns	ns
*mPFC*	14.4 ± 0.3	13.6 ± 0.2	14.1 ± 0.2	13.1 ± 0.3	**	ns	ns
*Insular cortex*	48.9 ± 0.3	48.5 ± 0.5	49.2 ± 0.7	48.9 ± 0.4	ns	ns	ns

### Early life stress effects on structural integrity

Next, we tested whether MD and the observed volumetric changes were accompanied by alterations in the structural integrity of the brain by comparing the relative fractions of white and grey matter within the cerebrum and each region calculated from the DTI-based FA and mean diffusivity maps (Tables 2 and 3). At the level of the whole brain, MD shifted the grey/white matter ratio as result of a lowered relative volume of white matter (F(1,33) = 5.54, p < 0.05), irrespective of the total brain volume (Table 2). No effect or interaction in response to MIF was found. The relative volume of grey matter was not affected (Table 3).

**Table 2:** Relative volumes of white matter content to whole cerebrum in the different ROIs (n = 10 per group) calculated from the DTI scan. Data represents percentage of total ROI volume in % (mean ± SEM). Data was analyzed using 2-way ANOVA with MD and mifepristone treatment as main factors. Statistical significance based on p-values for the main effects is presented in the last three columns: * p < 0.05, ** p < 0.01, ns = not significant.

White matter	Vehicle		Mifepristone		MD	Treatment	Interaction
**Rel. volume (%)**	**No MD**	**MD**	**No MD**	**MD**			
							
**Cerebrum**	13.3 ± 0.41	12.3 ± 0.22	12.5 ± 0.50	11.3 ± 0.60	*	ns	ns
**Corpus Callosum**	78.3 ± 1.39	77.5 ± 0.60	76.9 ±1.06	76.4 ± 1.34	ns	ns	ns
**Hippocampus**	11.0 ± 0.18	10.6 ± 0.32	10.2 ± 0.30	9.58 ± 0.38	p = 0.06	*	ns
**Amygdala**	3.92 ± 0.82	2.99 ± 0.36	3.09 ± 0.44	2.26 ± 0.51	ns	ns	ns
**dmStriatum**	2.40 ± 0.23	1.71 ± 0.13	2.14 ± 0.24	1.47 ± 0.22	**	ns	ns
**PFC**	1.23 ± 0.16	0.77 ± 0.14	0.78 ± 0.14	0.49 ± 0.14	*	*	ns
*OFC*	2.80 ± 0.29	2.10 ± 0.31	2.32 ± 0.38	1.28 ± 0.31	*	ns	ns
*mPFC*	1.57 ± 0.24	1.11 ± 0.18	0.89 ± 0.15	0.81 ± 0.20	ns	*	ns
*Insular cortex*	2.08 ± 0.27	1.37 ± 0.18	1.34 ± 0.36	0.98 ± 0.27	ns	ns	ns

**Table 3:** Relative volumes of grey matter content to whole cerebrum in the different ROIs (n = 10 per group) calculated from the DTI scan. Data represents percentage of total ROI volume in % (mean ± SEM). Data was analyzed using 2-way ANOVA with MD and mifepristone treatment as main factors. Statistical significance based on p-values for the main effects are described in the last three columns: * p < 0.05, ** p < 0.01, ns = not significant.

Grey matter	Vehicle		Mifepristone		MD	Treatment	Interaction
**Rel. volume (%)**	**No MD**	**MD**	**No MD**	**MD**			
							
**Cerebrum**	86.8 ± 0.73	87.0 ± 0.25	86.9 ± 0.50	87.6 ± 0.64	ns	ns	ns
**Corpus Callosum**	21.6 ± 1.39	22.5 ± 0.59	23.1 ± 1.07	23.6 ± 1.34	ns	ns	ns
**Hippocampus**	90.3 ± 0.98	89.3 ± 0.32	90.1 ± 0.44	91.2 ± 0.86	ns	ns	ns
**Amygdala**	97.0 ± 0.58	97.0 ± 0.36	97.1 ± 0.46	97.7 ± 0.51	ns	ns	ns
**dmStriatum**	97.8 ± 0.27	98.2 ± 0.13	97.9 ± 0.23	98.0 ± 0.35	ns	ns	ns
**PFC**	98.8 ± 0.16	99.0 ± 0.18	99.2 ± 0.15	99.3 ± 0.17	ns	ns	ns
*OFC*	97.4 ± 0.35	97.8 ± 0.34	97.6 ± 0.39	98.4 ± 0.38	ns	ns	ns
*mPFC*	98.2 ± 0.25	98.4 ± 0.25	98.9 ± 0.16	99.0 ± 0.20	ns	**	ns
*Insular cortex*	97.9 ± 0.35	98.2 ± 0.25	98.5 ± 0.36	98.4 ± 0.38	ns	ns	ns

To assess whether this was a general effect or whether there were specific changes in any of the ROIs, we also compared the relative grey and white volumes in specific brain regions. In the dorsomedial striatum (F(1,31) = 9.69, p < 0.01) and PFC (F(1,31) = 5.74, p < 0.05), MD significantly reduced relative white matter volume, while we found a trend in the hippocampus (p = 0.06). Subregion analysis showed that within the PFC, MD mainly affected the OFC (F(1,33) = 6.63, p < 0.05). Interestingly, while we did not find an interaction effect, MIF treatment itself reduced relative white matter volume in the PFC (F(1,31) = 5.44, p < 0.05), more specifically in the mPFC (F(1,33) = 5.92, p < 0.05). Similar results were found for hippocampus (F(1,33) = 5.28, p < 0.05) (Table 2). In the corpus callosum, insular cortex and amygdala we did not find changes in either the relative white or grey matter volumes (Tables 2 and 3).

We next aimed to identify whether the changes in white or grey matter fraction were accompanied by altered structural characteristics, using voxel-wise comparison of the DTI-based mean diffusivity, and fractional anisotropy (FA) or volumetric analysis within each ROI (Fig 1B and 1C, Table 4). This analysis did not reveal any significant differences between the groups (Table 4).

**Table 4:** Mean diffusivity (10^−3^ mm^2^/s) and FA per ROI calculated from the DTI scan. Data is expressed as mean ± SEM (n = 10 per group) and was analyzed using 2-way ANOVA with MD and mifepristone treatment as factors. Statistical significance of main effects is shown in the last three columns (ns = not significant).

	Vehicle		Mifepristone	
	No MD	MD	No MD	MD
**Mean Diffusivity**				
**Corpus Callosum**	0.20 ± 0.02	0.20 ± 0.01	0.20 ± 0.02	0.20 ± 0.01
**Hippocampus**	0.29 ± 0.01	0.29 ± 0.003	0.30 ± 0.01	0.29 ± 0.01
**Amygdala**	0.26 ± 0.01	0.26 ± 0.004	0.27 ± 0.01	0.26 ± 0.01
**dmStriatum**	0.26 ± 0.01	0.26 ± 0.004	0.26 ± 0.01	0.26 ± 0.01
**PFC**	0.25 ± 0.01	0.26 ± 0.01	0.26 ± 0.01	0.26 ± 0.01
*OFC*	0.24 ± 0.01	0.25 ± 0.01	0.24 ± 0.01	0.25 ± 0.01
*mPFC*	0.24 ± 0.01	0.25 ± 0.01	0.24 ± 0.01	0.25 ± 0.01
*Insular cortex*	0.24 ± 0.01	0.25 ± 0.01	0.25 ± 0.01	0.25 ± 0.01
**Mean FA**				
**Corpus Callosum**	0.52 ± 0.046	0.52 ± 0.012	0.52 ± 0.027	0.50 ± 0.045
**Hippocampus**	0.23 ± 0.004	0.24 ± 0.002	0.23 ± 0.002	0.23 ± 0.005
**Amygdala**	0.23 ± 0.007	0.22 ± 0.004	0.22 ± 0.005	0.22 ± 0.008
**dmStriatum**	0.18 ± 0.004	0.18 ± 0.002	0.18 ± 0.004	0.18 ± 0.005
**PFC**	0.20 ± 0.004	0.20 ± 0.003	0.19 ± 0.003	0.19 ± 0.005
*OFC*	0.20 ± 0.004	0.20 ± 0.003	0.20 ± 0.002	0.20 ± 0.005
*mPFC*	0.20 ± 0.005	0.20 ± 0.004	0.19 ± 0.004	0.19 ± 0.004
*Insular cortex*	0.20 ± 0.004	0.20 ± 0.003	0.19 ± 0.004	0.19 ± 0.006

Altogether these data indicate that MD specifically affected the ratio between white and grey matter, by decreasing white (relative to gray) matter volume in the cerebrum as a whole, and more specifically in the dorsomedial striatum and PFC. This was however not accompanied by significant changes in the regional FA or mean diffusivity.

## Discussion

In the present study we investigated the effect of early life stress (by 24 h MD at PND 3) on the structural integrity of the adult rodent brain. We demonstrate that MD was associated with relatively modest regional volumetric changes and shifts in grey:white matter ratios, none of which were normalized by transient blockade of glucocorticoid receptors during adolescence.

### Experimental paradigm

In the current study we chose to study behaviorally naïve animals to exclude any possible confounding task-inducing effects on structural integrity and brain volume. Although we did not examine any stress-sensitive parameters (e.g. corticosterone level) in the presently used batch of animals, we have no reason to doubt the effectiveness of the MD paradigm. Earlier this procedure was shown to be sufficiently severe to significantly reduce body weight, increase levels of corticosterone [34], alter glucocorticoid receptor expression (in the mPFC) and affect glutamatergic transmission (Loi et al. unpublished observation; study performed in parallel). Moreover, this paradigm was also shown to severely impair the behavioral performance of rats, as shown e.g. by deficits in memory formation [34].

Although some brain regions seem sensitive to MD, MIF was not effective in normalizing the effects of early life stress on structure. The discrepancies between the current and earlier studies cannot be explained by assuming that MIF in general is ineffective in targeting structural plasticity. Earlier, it was reported that brief treatment with MIF is able to ameliorate the effects caused by exposure to chronic stress in adulthood on rat hippocampal structural plasticity [35]. Moreover, a recent study demonstrated that brief MIF treatment during early adolescence can fully reverse the behavioral phenotype induced by early life stress [27]. The treatment window in that study was selected based on the high sensitivity of the brain and the HPA axis during this critical time window [26]. MIF is not only effective in ameliorating behavioral effects of stress in rodents but also in humans, where this compound has shown its effectiveness in alleviating the symptoms of major depression and bipolar disorder [36,37]. We cannot exclude, however, that the ineffectiveness of MIF to normalize the subtle effects of MD in the current study was related to the selected MIF administration paradigm.

### Regional structural effects of maternal deprivation

Interestingly, the volume of the prefrontal cortex was found to be reduced after MD. More specifically, the medial PFC and the orbitofrontal cortex were identified as the subregions with the most pronounced volumetric reduction, although the effects were relatively small. This is in line with other studies in humans which report volumetric reduction in the medial prefrontal cortex [38] and orbitofrontal cortex [39] in stress-related mental illnesses such as post-traumatic stress disorder and major depression. Particularly the finding in the mPFC fits with its role in regulating the endocrine stress response and the regulation of adaptive coping responses to stress [40]. Additionally, regions of the PFC such as the orbital, medial and cingulate cortex are strongly involved in the allostatic processes [41]. Furthermore, as shown in earlier rat studies, the mPFC appears to be a major target area of glucocorticoids, since three weeks of corticosterone treatment (in rats) led to dramatic changes at the microscopic structural level, such as dendritic tree atrophy [42] and reduced spine density [43]. Prefrontal cortex dendritic remodeling due to stress is also hypothesized to be involved in deficits in attentional control that are seen in stress-related pathologies [44]. Interestingly, rodent studies employing chronic stress in adulthood showed opposite effects in the mPFC compared to OFC. While reduction in dendritic complexity was shown in the former area–which would be compatible with a reduced volume- increased complexity was described in the latter. In our study we did not observe opposite but rather comparable effects of MD in these two areas. Obviously, prolonged corticosterone-overexposure in adulthood does not necessarily induce similar effects as a brief period of MD early in life. Moreover, although changes in the microstructure such as dendritic complexity may be identified from altered FA or mean diffusivity values, previous MRI studies on chronic stress in rodents have shown that they cannot be directly translated into volumetric changes [29]. Yet, the here reported effects on volume, particularly in these two PFC ROIs, are in line with volumetric studies in humans.

Notably, while MD modestly yet specifically reduced PFC volume we did not find concomitant compensatory changes in other regions such as the ventricles. This may be attributed to the differences in susceptibility to early life stress in relation to timing of the model and the developmental rate of different brain regions. In that respect, MD did result in a slightly smaller cerebrum volume, though not statistically significant. This however may indicate that brain development may be impaired in some regions overall but not in those that were specifically included in this study. The present design also did not lead to extensive volumetric changes in areas involved in memory formation (such as the hippocampus) or in the control of emotions (e.g. the amygdala). The human literature on volumetric changes in the hippocampus and amygdala after adverse conditions, however, is quite diverse [20], both in the adult and younger population. For instance, exposure to aggressive maternal behavioral did not lead to changes in hippocampal volume when assessed during adolescence [19,45] whereas a decrease was reported in other studies [46,47]. Others using rodent models for chronic stress early or later in life have also found more robust changes in hippocampal volume or integrity [48,49]. The differences in outcomes may be attributed to discrepancy in age of the animal, type and timing of the stress model which may impact on different developmental windows of susceptible brain areas and circuits.

Concerning the amygdala, the same inconsistency has been found in human studies, where some report a link between early life stressors and increased amygdaloid volume [19] whereas other studies show either a reduction [47] or no differences at all [50–52]. Chronic stress exposure in adult rodents generally resulted in an enlarged amygdala volume [53,54]. As pointed out above though, chronic stress in adulthood is not necessarily expected to induce the same effect as 24h maternal deprivation early in life.

In addition to these volumetric changes, voxel-based morphometry revealed that exposure to maternal deprivation resulted in aberrant white matter volume. A wide range of mental illnesses, including neurodevelopmental and cognitive disorders, have been related with white and grey matter defects; and some authors support the theory that myelin abnormalities affect processing and cognition [55]. In line with this, results from human neuroimaging studies reported an association between early life stress and alteration in the white and grey matter volume in stress-sensitive brain areas in adulthood [21,22,47]. In our study, MD-induced decreases in white matter fraction were found while grey matter fractions seemed largely unaffected. This decrease in relative white matter content was apparent for the whole cerebrum but we also report abnormalities at the level of single brain areas such as the PFC and dorsomedial striatum and at trend level for the hippocampus. In that respect, the volumetric decrease in PFC may be attributed to a smaller white matter fraction in this brain. Although the underlying mechanism is unclear, MD has been shown to increase cell death in white matters tracts of the cortex and dentate gyrus [56], which could affect connectivity processes related to behavior.

In the hippocampus, we found a trend towards decreased relative white matter volume. Although not significant, this finding is in line with most literature on human hippocampus. Frodl and colleagues reported a reduction in hippocampal white matter in patients suffering from major depression who also experienced emotional neglect during childhood [16,57].

In the current study we did not observe major changes in grey matter volume in the cerebrum or ROIs which was a somewhat unexpected finding. Human studies have shown that childhood maltreatments are associated with a reduction in grey matter in several brain areas including subregions of the striatum, PFC and hippocampus [58,59]. It should be noted that the changes in grey and white volume fractions that we report were not accompanied by significant changes in diffusion parameters measured from subsequent DTI analysis. This suggests that the structural integrity of these brain regions remained largely preserved at macroscopic level. Alternatively, a recent paper by Yan et al showed that maternal maltreatment specifically affect functional connectivity in the amygdala-PFC pathway during development [60]. As we were not able to perform functional analysis on post-mortem tissue it is possible that the MD procedure is more likely to affect functional connectivity even though we did not observe any changes on structural plasticity.

### Concluding remarks

We here present one of the few studies where the effects of early life stress are investigated at the level of macroscopic structure and structural integrity in the rat brain, using high-resolution magnetic resonance imaging. We only observed subtle structural effects in areas expected to be sensitive to MD, based on earlier studies involving early life stress or chronic stress in adulthood [29,34]. Although the within-group variation was quite low, we cannot exclude that more pronounced effects would be discernable when applying a within-subjects design, rather than the current cross-sectional between-subjects approach; this may be one of the explanations why e.g. MRI studies investigating effects of chronic stress reported no structural changes in between-subjects comparisons [29], whereas such changes did show up in within-subjects comparisons [61]. Clearly, our (post-mortem) high-resolution anatomical investigations are not compatible with repeated within-subjects measurements.

Future studies would need to examine whether alterations in morphology, synaptic plasticity and reduction in volume of specific brain areas are also related to altered connectivity in the rodent brain. Clearly, more studies where the effects of stress early in life (in rodents) are consecutively assessed in both behavioral, macroscopic and connectivity outcome are necessary to improve our understanding of how early life stress can lead to abnormalities in the developing brain. Given the bias of specific mental illnesses towards the female population, such studies should also be carried out in females exposed to early life stress conditions.
